# Supplementary material for: Determinants of intention to leave among nurses and physicians in a hospital setting during the COVID-19 pandemic: A systematic review and meta-analysis
Source: PLoS One. 2024 Mar 14;19(3):e0300377. doi: 10.1371/journal.pone.0300377 (PMC10939201; doi:10.1371/journal.pone.0300377)
Supplement: S2 Table — (DOCX) [file pone.0300377.s002.docx]

S2 Table. Literature search

PubMed search

| Domain | Exposure | Outcome |
| --- | --- | --- |
| Healthcare professionals | **COVID-19** | **Job retention/turnover** |
| **(((((((((((((((((((("Health Personnel"[Mesh]) OR ("Health Personnel")) OR ("healthcare professional*")) OR ("health care professional*")) OR ("healthcare worker*")) OR ("health care worker*")) OR ("healthcare provider*")) OR ("health care provider*")) OR ("Healthcare staff")) OR ("Health care staff")) OR ("Health worker*")) OR ("Nurses"[Mesh])) OR (Nurse)) OR (Nursing)) OR ("Nurse's Role"[Mesh])) OR ("Nursing Staff, Hospital"[Mesh])) OR ("Physicians"[Mesh])) OR (Physician*)) OR ("medical specialist*"))** | **(((((("COVID-19"[Mesh]) OR (Covid-19)) OR ("SARS-CoV-2"[Mesh])) OR ("SARS-CoV-2")) OR (covid-19 pandemic)) OR ("severe acute respiratory syndrome coronavirus 2"))** | **(((((((((((((((("Personnel Turnover"[Mesh]) OR ("Personnel Turnover*")) OR ("Employee Turnover*")) OR ("Personnel Retention*")) OR (job retention)) OR ("retention rates")) OR ("turnover intention*")) OR ("intention to leave")) OR ("intention to quit")) OR ("intent to quit")) OR ("intent to leave")) OR ("staff turnover*")) OR ("quit the job")) OR ("retaining personnel")) OR ("intention to stay") OR (retention))))** |

Embase Search

| Domain | Exposure | Outcomes |
| --- | --- | --- |
| Healthcare professionals | **COVID-19** | **Job retention/turnover** |
| 'medical specialist'/exp OR 'medical specialist' OR 'physician'/exp OR physician OR 'nurse role' OR (('nurse'/exp OR nurse) AND ('role'/exp OR role)) OR 'nursing staff'/exp OR 'nursing staff' OR (('nursing'/exp OR nursing) AND ('staff'/exp OR staff)) OR 'nurse'/exp OR nurse OR 'health worker'/exp OR 'health worker' OR (('health'/exp OR health) AND ('worker'/exp OR worker)) OR  healthcare AND staff OR healthcare AND provider OR healthcare AND worker OR health AND care AND worker OR 'health care professional' OR health AND care AND professional OR 'health care personnel' OR 'health personnel'/exp OR 'health personnel' OR (('health'/exp OR health) AND ('personnel'/exp OR personnel)) | 'coronavirus disease 2019'/exp OR 'coronavirus disease 2019' OR 'severe acute respiratory syndrome coronavirus 2'/exp OR 'severe acute respiratory syndrome coronavirus 2' OR (severe AND acute AND ('respiratory'/exp OR respiratory) AND ('syndrome'/exp OR syndrome) AND ('coronavirus'/exp OR coronavirus) AND ('2'/exp OR 2)) OR 'covid pandemic' OR (('covid'/exp OR covid) AND ('pandemic'/exp OR pandemic)) OR  'severe acute respiratory syndrome coronavirus 2'/exp OR 'severe acute respiratory syndrome coronavirus 2' OR 'sars cov 2'/exp OR 'sars cov 2' OR 'covid 19'/exp OR 'covid 19' | 'retention'/exp OR retention OR 'intention to stay' OR (('intention'/exp OR intention) AND to AND stay) OR 'retaining personnel' OR (retaining AND ('personnel'/exp OR personnel)) OR 'quit the job' OR (quit AND the AND ('job'/exp OR job)) OR 'staff turnover'/exp OR 'staff turnover' OR 'intention to quit' OR (('intention'/exp OR intention) AND to AND quit) OR 'intention to leave' OR (('intention'/exp OR intention) AND to AND leave) OR 'turnover intention'/exp OR 'turnover intention' OR 'job retention'/exp OR 'job retention' OR (('job'/exp OR job) AND ('retention'/exp OR retention)) OR 'personnel retention' OR (('personnel'/exp OR personnel) AND ('retention'/exp OR retention)) OR 'employee turnover' OR (('employee'/exp OR employee) AND ('turnover'/exp OR turnover)) OR 'personnel turnover'/exp OR 'personnel turnover' OR (('personnel'/exp OR personnel) AND ('turnover'/exp OR turnover)) OR 'personnel management'/exp OR 'personnel management' |

CINAHL Search

| Domain | Exposure | Outcomes |
| --- | --- | --- |
| Healthcare professionals | **COVID-19** | **Job retention/turnover** |
| (MH "Health Personnel+") OR "Healthcare professional" OR "Healthcare professionals" OR "Health care professionals" OR "Health care professional" OR "healthcare worker" OR "healthcare workers" OR "health care worker" OR "health care workers" OR "healthcare providor" OR "healthcare providors" OR "healthcare provider" OR "healthcare providers" OR "healthcare staff" OR "health care staff" OR "health worker" OR "health workers" OR (MH "Nurses+") OR "nurse" OR "nursing" OR (MH "Nursing Staff, Hospital") OR (MH "Physicians+") OR "physician" OR "doctor" OR "medical specialist" OR "medical specialists" | (MH "COVID-19+") OR (MH "SARS-CoV-2") OR MH "COVID-19 Pandemic") OR "covid-19" OR "severe acute respiratory syndrome coronavirus 2" OR "SARS-CoV-2" | (MH "Personnel Turnover") OR "personnel turnover" OR "employee turnover" OR (MH "Personnel Retention") OR "job retention" OR "retention rates" OR "turnover intention" OR "intention to leave" OR "intention to quit" OR "intent to leave" OR "staff turnover" OR "quit the job" OR "retaining personnel" OR (MH "Employment Termination") OR "intention to stay" OR "retention" OR "attrition" |

Web of Science SEARCH

| Domain | Exposure | Outcomes |
| --- | --- | --- |
| Healthcare professionals | **COVID-19** | **Job retention/turnover** |
| ((((((((((((((((((ALL=(Health Personnel)) OR ALL=(healthcare professional )) OR ALL=(health care professional )) OR ALL=(healthcare worker)) OR ALL=(health care worker)) OR ALL=(healthcare provider)) OR ALL=(health care provider)) OR ALL=(healthcare staff)) OR ALL=(health care staff)) OR ALL=(health worker)) OR ALL=(nurses)) OR ALL=(nurse)) OR ALL=(nursing)) OR ALL=(nursing staff)) OR ALL=(physician)) OR ALL=(physicians)) OR ALL=(doctor)) OR ALL=(medical specialist)) OR ALL=(medical specialists) | (((ALL=(COVID-19)) OR ALL=(SARS-CoV-2)) OR ALL=(covid-19 pandemic)) OR ALL=(severe acute respiratory syndrome coronavirus 2) | ((((((((((((((ALL=(Personnel Turnover)) OR ALL=(Employee turnover)) OR ALL=(Personnel Retention)) OR ALL=(job retention)) OR ALL=(retention rates)) OR ALL=(turnover intention )) OR ALL=(intention to leave)) OR ALL=(intention to quit)) OR ALL=(intent to quit)) OR ALL=(intent to leave)) OR ALL=(staff turnover)) OR ALL=(quit the job)) OR ALL=(retaining personnel )) OR ALL=(intention to stay)) OR ALL=(retention) |

Search 25/1/2024 1176 hits
