# Supplementary material for: Determinants of intention to leave among nurses and physicians in a hospital setting during the COVID-19 pandemic: A systematic review and meta-analysis
Source: PLoS One. 2024 Mar 14;19(3):e0300377. doi: 10.1371/journal.pone.0300377 (PMC10939201; doi:10.1371/journal.pone.0300377)
Supplement: S3 Table — (DOCX) [file pone.0300377.s003.docx]

S3 Table. Data-extraction and quality assessment summary of included records for systematic review.

| **First author (year)** | **Type of study** | **Country** | **Sample** | **Sample size** | **Measurement** | **Determinants** | **Quality Assessment^a^** |
| --- | --- | --- | --- | --- | --- | --- | --- |
| Abd-Ellatif (2021) | Cross-sectional | Egypt | Physicians | 411 | Intention to leave | Job satisfaction, fear of COVID-19 | 4/4 |
| Ahmed (2022) | Cross-sectional | United Arab Emirates | Nurses and Physicians | 538 | Intention to leave | Marital status, organizational support | 4/4 |
| Al Hadid (2022) | Cross-sectional | Jordan | Nurses | 300 | Intention to stay | Financial refuse | 4/5 |
| Alblihed (2022) | Cross-sectional | Saudi Arabia | Nurses | 392 | Intention to leave | Burnout | 5/5 |
| Alnaeem (20220 | Cross-sectional | Jordan | Nurses and physicians | 57 | Intention to leave the current job | Marital status, night shifts, fear of COVID-19, organizational support | 4/4 |
| Çetin Aslan (2022) | Cross-sectional | Turkey | Nurses and physicians | 373 | Intention to leave | Organizational support, macro-control perception | 4/4 |
| Chen (2021) | Cross-sectional | Taiwan | Nurses | 333 | Intention to stay in the current job | Taking care of COVID-19 patients, course to provide pandemic care, clinical stress, willingness to provide services | 5/5 |
| Chen 2022) | Cross-sectional | Taiwan | Nurses and physicians | 1359 | Intention to leave the current job | Age, job stress, night shifts, lengths of services, amount of working hours, taking care of COVID-19 patients, high perceived risk of COVID-19, supportive administration/management, organizational support | 5/5 |
| Cole (2021) | Cross-sectional | United States | Nurses | 111 | Intention to leave | Gender, age, marital status, professional health, patient assignments, shortage of PPE, patients’ acuity, psychosocial support | 4/4 |
| De Cordova (2022) | Cross-sectional | United States | Nurses | 3030 | Intention to leave | Burnout, shortage of PPE, lack of confidence in available PPE, lack of confidence in adequacy of PPE | 3/5 |
| Ekingen (2023) | Cross-sectional | Turkey | Nurses | 486 | Intention to leave | Gender, heavy workload, working department, district work, fear of COVID-19 | 4/5 |
| Fasih Far (2022) | Cross-sectional | Iran | Nurses | 299 | Intention to leave | Burnout, job satisfaction, gender of patients | 3/5 |
| Fronda (2022) | Cross-sectional | Philippines | Nurses | 687 | Intention to leave | Coping skills, coronaphobia, social support | 5/5 |
| Gherman (2022a) | Experimental study | Romania | Nurses | 634 | Intention to leave | Self-disclosure, burnout, autonomy satisfaction, competence satisfaction, relatedness satisfaction, work engagement, supervisor support | 4/4 |
| Gherman (2022b) | Quasi-experimental | Romania | Nurses | 463 | Intention to leave | Burnout, COVID-19 discrimination, Potentially Morally Injuries Events PMIE’s | 3/4 |
| Gümüşsoy (2022) | Descriptive | Turkey | Nurses | 371 | Intention to leave the profession | Burnout, day shifts, change of duty during pandemic period, length of service, salary, insufficient amount of medical personal | 5/5 |
| Habibzadeh (2022) | Cross-sectional | Iran | Nurses | 295 | Intention to leave | Bullying, horizontal violence | 5/5 |
| Hanum (2022) | Cross-sectional | China | Nurses | 1425 | Intention to stay | Age, job satisfaction, professional identity, length of service, job title | 5/5 |
| Hwang (2022) | Descriptive | Korea | Nurses | 207 | Intention to leave | Job stress, quality of work life | 5/5 |
| Jing (2022) | Cross-sectional | China | Nurses | 443 | Intention to leave | PTSD | 3/4 |
| Jiwnani (2021) | Cross-sectional | India | Physicians | 1041 | Intention to leave | Increasing domestic responsibilities | 4/5 |
| Jo (2021) | Cross-sectional | Japan, the Republic of Korea, Turkey and the United States | Nurses | 904 | Intention to leave the profession | Resilience | 4/5 |
| Karimi (2022) | Cross-sectional | Iran | Nurses | 170 | Intention to leave | Depersonalization, economic situation, ability to take a rest, organizational commitment | 4/4 |
| Khattak (2021) | Cross-sectional | Pakistani | Nurses | 380 | Intention to leave | Moral distress, secondary trauma, fear of COVID-19 | 3/5 |
| Kim (2020) | Cross-sectional | Korea | Nurses | 377 | Intention to leave the current job | Age, marital status, salary, nursing position, experience, encouragement through mass media | 5/5 |
| Kuriyama (2023) | Cross-sectional | Japan | Nurses and physicians | 936 | Intention to leave | Burnout, conflicts with colleagues, organizational support, psychosocial resilience, pride to work as a critical professional | 3/4 |
| Labrague (2021a) | Cross-sectional | Philippines | Nurses | 259 | Intention to leave the current job,  intention to leave the profession | Psychosocial resilience, COVID-19-associated discrimination | 4/4 |
| Labrague (2021b) | Cross-sectional | Philippines | Nurses | 270 | Intention to leave the current job,  intention to leave the profession | Compassion fatigue | 5/5 |
| Labrague (2021c) | Cross-sectional | Philippines | Nurses | 261 | Intention to leave the current job,  intention to leave the profession | Fear of COVID-19 | 4/4 |
| Lavoie-Tremblay (2022) | Cross-sectional | Canada | Nurses | 1705 | Intention to leave the current job, intention to leave the profession | Years of experience, professional competence, feeling overwhelmed during pandemic, chronic fatigue, job satisfaction, taking care of COVID-19 patients, self-infected or team member infected with COVID-19, situation at work, leadership | 4/5 |
| Linzer (2022) | Cross-sectional | United States | Nurses and physicians | 20627 | Intention to leave | Job stress, poor alignment, teamwork, work control | 5/5 |
| Lou (2021) | Cross-sectional | Canada | Nurses and physicians | 119 | Intention to quit | Hospital resources, psychological distress, burnout | 5/5 |
| Lou (2022) | Cross-sectional | Canada | Nurses and physicians | 119 | Intention to quit | Burnout | 3/4 |
| Mirzaei (2021) | Cross-sectional | Iran | Nurses | 479 | Intention to leave | Marital status, general health, PTSD, decision latitude, job demands, job insecurity, job strain, organizational support, social support | 4/5 |
| Nashwan (2021) | Cross-sectional | Qatar | Nurses | 512 | Intention to leave | Years of experience, length of service, COVID-19 | 5/5 |
| Ohue (2021) | Cross-sectional | Japan | Nurses | 56 | Intention to resign | Burnout, anxiety disorder, depression | 3/4 |
| Ohue (2023) | Cross-sectional | Japan | Nurses | 56 | Intention to quit the job | Burnout | 3/4 |
| Piotrowski (2022) | Cross-sectional | Poland | Nurses and physicians | 441 | Intention to leave current the job | Job satisfaction, job stress, resilience | 4/4 |
| Pahleven Sharif (2023) | Cross-sectional | Iran | Nurses | 305 | Intention to leave | Job satisfaction, organizational support | 3/4 |
| Rafiq (2022) | Cross-sectional | Pakistani | Nurses | 520 | Intention to leave | Length of service, trusted climate, job embeddedness | 3/4 |
| Raso (2021) | Cross-sectional | United States | Nurses | 5088 | Intention to leave the current job, intention to leave the profession | Age, years of experience, role, working department | 4/5 |
| Romero-García (2022) | Cross-sectional | Spain | Nurses and physicians | 434 | Intention to leave the current job | Moral distress, working department | 4/5 |
| Sert-Ozen (2023) | Cross-sectional | Turkey | Physicians | 201 | Intention to leave the profession | Emotional exhaustion, moral injuries | 4/5 |
| Shah (2021) | Cross-sectional | Pakistani | Nurses | 301 | Intention to leave | Emotional exhaustion, job stress, state anger | 3/4 |
| Shayestehazar (2022) | Descriptive | Iran | Nurses | 172 | Intention to leave the current job | Years of experience, organizational commitment | 3/4 |
| Sheppard (2022) | Cross-sectional | United States | Nurses | 129 | Intention to leave the current job | Moral distress | 4/5 |
| Sipos (2023) | Cross-sectional | Hungary | Nurses and physicians | 205 | Intention to leave the current job, intention to leave the profession | Depersonalization, emotional exhaustion, personal accomplishment | 4/5 |
| Sonis (2022) | Cross-sectional | United States | Physicians | 810 | Intention to leave the profession | Duty shifts, moral distress | 3/5 |
| Sun (2023) | Cross-sectional | China | Nurses | 374 | Intention to leave | Fatigue | 5/5 |
| Wang (2022) | Cross-sectional | Taiwan | Nurses | 598 | Intention to leave | Gender, age, emotional intelligence | 4/5 |
| Wibowo (2022) | Cross-sectional | Indonesia | Nurses | 188 | Intention to leave | Mindful and empathetic leadership, self-regulation | 5/5 |
| Zeiher (2022) | Cross-sectional | China | Nurses | 56 | Intention to leave the current job | Practice environment | 4/4 |
| Zeng (2022) | Cross-sectional | China | Nurses | 2299 | Intention to leave | Age, marital status, children, years of experience, day shifts, salary, amount of working hours a week, professional title, position, working department, relation with immediate leader, support from family | 4/4 |
| Zhou (2022) | Cross-sectional | China | Nurses | 650 | Intention to leave | Job alternatives, organizational justices | 5/5 |

^a^The quality assessment was conducted using the Mixed Methods Appraisal Tool (MMAT) (version 2018). ‘Yes’ counted for one point and ‘No’ for zero points. In case a quality criterion was answered with ‘cannot tell’, more information was needed to give a legit answer in terms of ‘yes’ or ‘no’[70]. Therefore, this criterion is not included in the overall score.
