# Supplementary material for: Determinants of intention to leave among nurses and physicians in a hospital setting during the COVID-19 pandemic: A systematic review and meta-analysis
Source: PLoS One. 2024 Mar 14;19(3):e0300377. doi: 10.1371/journal.pone.0300377 (PMC10939201; doi:10.1371/journal.pone.0300377)
Supplement: Attachment — Submitted filename: review_PONE_meta.docx [file pone.0300377.s004.docx]

Thank you for giving me the opportunity to review this systematic review and metanalysis about the intention to leave of healthcare workers working in hospital setting during Covid-19 pandemic.

The systematic review presented in this paper identifies six key themes of determinants impacting nurses' and physicians' intention to leave during the COVID-19 pandemic, shedding light on critical factors affecting retention.

The meta-analysis results reveal a substantial prevalence of intention to leave among healthcare professionals, highlighting the urgent need for hospital administrators to address this concerning trend.

This study underscores the multifaceted nature of determinants influencing healthcare workers' retention, emphasizing the role of fear of COVID-19, personal characteristics, and organizational support in shaping their career intentions.

The findings presented in this paper provide valuable insights for developing targeted strategies to retain nurses and physicians in hospital settings during the ongoing pandemic, with a focus on addressing both systemic and individual factors.

Methodologically the paper respects the PRISMA Checklist.

Please correct minor typo in the abstract: ‘’ Fourty-seven’’ and ‘’Metaanalysis’’

P 20 This increases the chanhe of creating

And please check the references, some of them used incorrect abbreviation of journal title.

5. Boniol M, Kunjumen T, Nair TS, et al. The global health workforce stock and distribution in 2020 and 2030: a threat to equity and ‘universal’ health coverage? BMJ Glob Heal;

[29] Alotibi, AM. Alalwan, FH. Alabdallah A. Factors Associated with Nurses Intention to Leave During COVID-19Pandemic: Literatue Review. Saudi J Nurs Heal Care 2023; 6: 159–167.

[60] Petrișor C, Breazu C, Doroftei M, et al. Association of Moral Distress with Anxiety, Depression, and an Intention to Leave among Nurses Working in Intensive Care Units during the COVID-19 Pandemic. Healthc (Basel, Switzerland); 9. Epub ahead of print October 2021. DOI: 10.3390/healthcare9101377.

[107] Jing C, Feng-Hong Z, Yi-Yan W. An investigation of the incidence of post-traumatic stress disorder, turnover intention and psychological resilience among medical staff in a public hospital in China during the outbreak of the omicron variant in the COVID-19 pandemic in2022. Front psychiatry 2022; 13: 999870.
